# Klebsiella-Induced Necrotizing Neck Infection in a Patient With Diabetes: A Case Report

**DOI:** 10.7759/cureus.78244

**Published:** 2025-01-30

**Authors:** Abdulrahman Alosaimi, Murad A Banjar, Walaa A Felemban, Khalid A Abulnassr, Yasser A Sabbagh

**Affiliations:** 1 Department of Otolaryngology-Head and Neck Surgery, Ohud Hospital, Madinah, SAU; 2 College of Medicine, Rayan Medical College, Madinah, SAU

**Keywords:** diabetic complications, klebsiella pneumoniae, neck infections, necrotizing soft tissue infections, surgical debridement

## Abstract

Necrotizing infections of the head and neck are uncommon, aggressive, and potentially fatal if not treated promptly. These infections are particularly prevalent among immunocompromised individuals, such as people with diabetes, due to impaired immune response and delayed healing. We present a 45-year-old male with uncontrolled diabetes who had a five-day history of progressive left-sided neck swelling and pain. Imaging studies revealed an extensive necrotizing infection of the submandibular, parotid, and parapharyngeal carotid spaces. The condition was managed with intravenous antibiotics, ultrasound-guided aspiration, surgical drainage, extensive debridement, and skin grafting. The isolated microorganism was Klebsiella pneumoniae. After three weeks of management, the patient was discharged without impairment or recurrence. This case underscores the significance of early diagnosis, proper antibiotic use, timely surgery, and collaboration in managing neck space necrotizing infections to prevent complications in high-risk patients.

## Introduction

Necrotizing soft tissue infections (NSTIs) rank among the most challenging and deadly infections, destroying skin, subcutaneous tissue, and fascia while presenting signs of systemic toxicity [1,2]. NSTIs are most commonly observed in the extremities but infrequently in the neck [3-5]. In this regard, the neck poses a specific danger due to its complex anatomy involving vital vascular, neural, and respiratory components. Thus, infections in this area put the patient at risk of permanent disabilities as vital functions can be compromised, and the infection can extend to adjacent regions, including the mediastinum [6].

One factor that makes NSTIs subtle is their ability to spread along fascial planes, as symptoms are often masked until the later stages [7]. Infections in the cervical region pose additional challenges due to diagnostic delays. These signs and symptoms are frequently vague and may resemble less severe conditions, such as cellulitis or superficial abscesses. This highlights the need for physicians to have a low-threshold suspicion for such a condition, particularly in high-risk populations like patients with diabetes [8].

Diabetes severely disrupts the functions of the neutrophils, including chemotaxis and phagocytosis [9]. In addition, microvascular injury impedes the delivery of immune cells and antibiotics to their target tissues, thereby augmenting the chances of infection resolution. In such patients, even trivial infections may progress into life-threatening necrosis or more severe sequelae involving deeper structures [10].

Klebsiella pneumoniae infections are particularly challenging due to the bacterium’s virulence factors, such as hypermucoviscosity and biofilm formation [11]. While it is known to cause severe infections, its involvement in necrotizing neck infections is rare, making this case clinically significant [12].

This case adds to the limited literature on necrotizing neck infections, highlighting the importance of early intervention. It stands out due to the patient’s uncontrolled diabetes, rapid infection progression, and successful multidisciplinary management, offering valuable insights for similar cases.

## Case presentation

A 45-year-old male with uncontrolled diabetes mellitus presented with complaints of left-side neck swelling that started two days before presentation. The swelling had worsened over five days, along with pain in that area. The patient denied systemic symptoms such as fever, shortness of breath, or upper respiratory tract infections. His diabetes was treated with oral hypoglycemic medication.

Clinical examination

**Figure 1:**
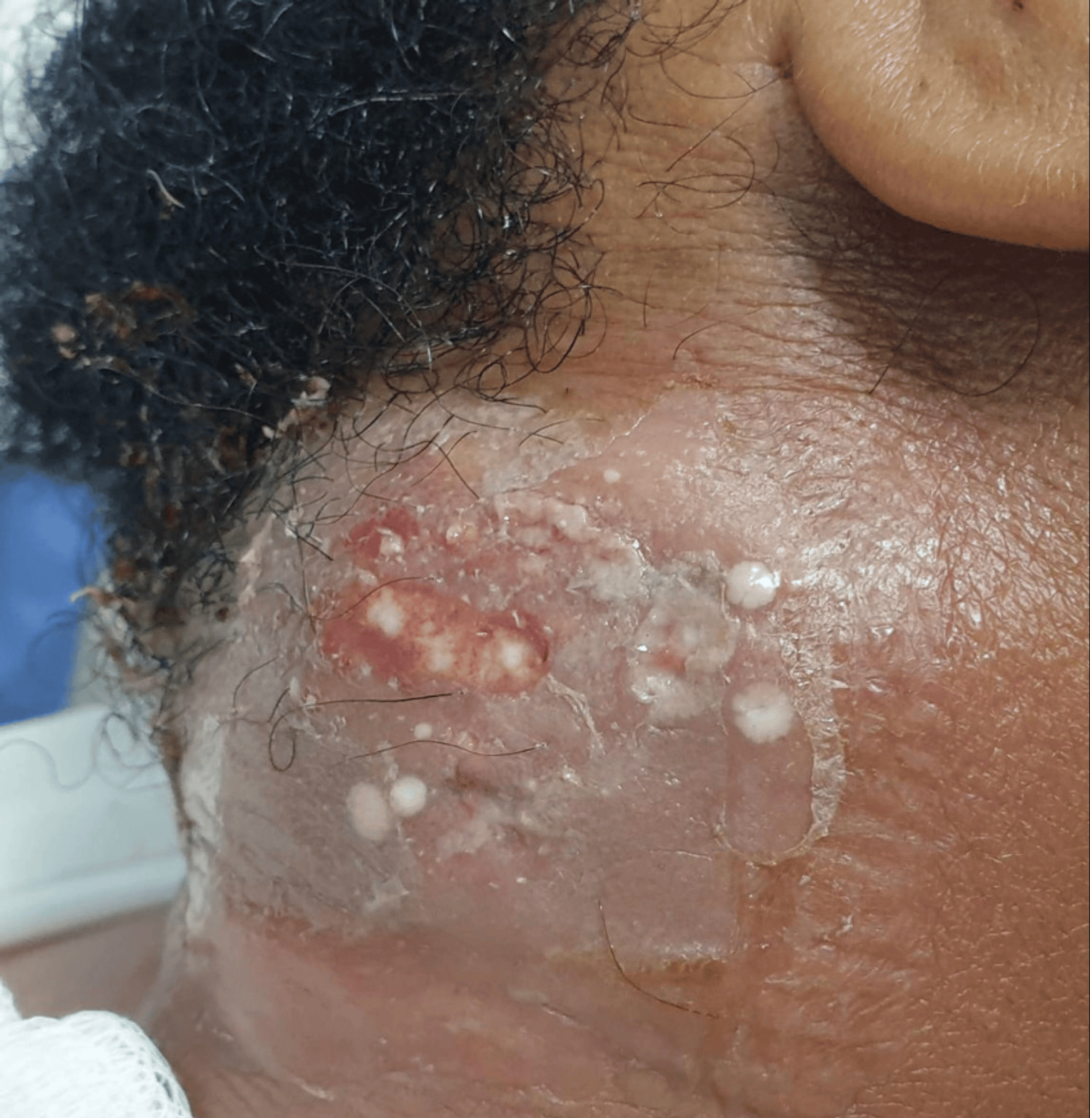
Neck swelling and erythema five days after starting antibiotics and seven days from onset, showing a limited response.

The patient was hemodynamically stable but exhibited poor dental hygiene. A firm, tender, and non-fluctuant 8 × 8 cm mass was noted in the left infra-auricular region, accompanied by overlying erythema and hotness (Figure 1). There was no lymphadenopathy, salivary gland discharge, or airway compromise. The oropharyngeal examination revealed a central uvula with no signs of bulging in the tonsils. The maxillofacial team excluded odontogenic or salivary gland sources of infection.

Investigations

The lab workup showed leukocytosis, high C-reactive protein (CRP) levels, and an increased erythrocyte sedimentation rate (ESR). On admission, the patient also had fluctuating blood glucose levels, yet he managed to normalize them after consulting the medical team. Table 1 summarizes the patient’s laboratory investigations, including inflammatory markers, glycemic control, and culture results, to highlight the diagnostic findings and guide clinical management.

**Table 1:** Summary of laboratory investigations highlighting inflammatory markers, glycemic control, and culture results.

Parameter	Patient's Value	Reference Range	Comments
White Blood Cell Count (WBC)	15,000 /mm³	4,000-11,000 /mm³	Elevated, indicating an inflammatory response.
C-reactive Protein (CRP)	120 mg/L	<5 mg/L	Significantly elevated, consistent with acute infection.
Erythrocyte Sedimentation Rate (ESR)	50 mm/hr	0-20 mm/hr	Elevated, reflecting ongoing inflammation.
Random Blood Glucose	250 mg/dL	70-140 mg/dL	Elevated, indicating poor glycemic control.
Hemoglobin A1c (HbA1c)	10.2%	<6.5%	Indicates poor long-term glycemic control.
Creatinine	1.2 mg/dL	0.6-1.2 mg/dL	Within normal limits.
Albumin	3.5 g/dL	3.4-5.4 g/dL	Normal.
Culture Result	Klebsiella pneumoniae	-	Pathogen identified from purulent aspirate.

**Figure 2:**
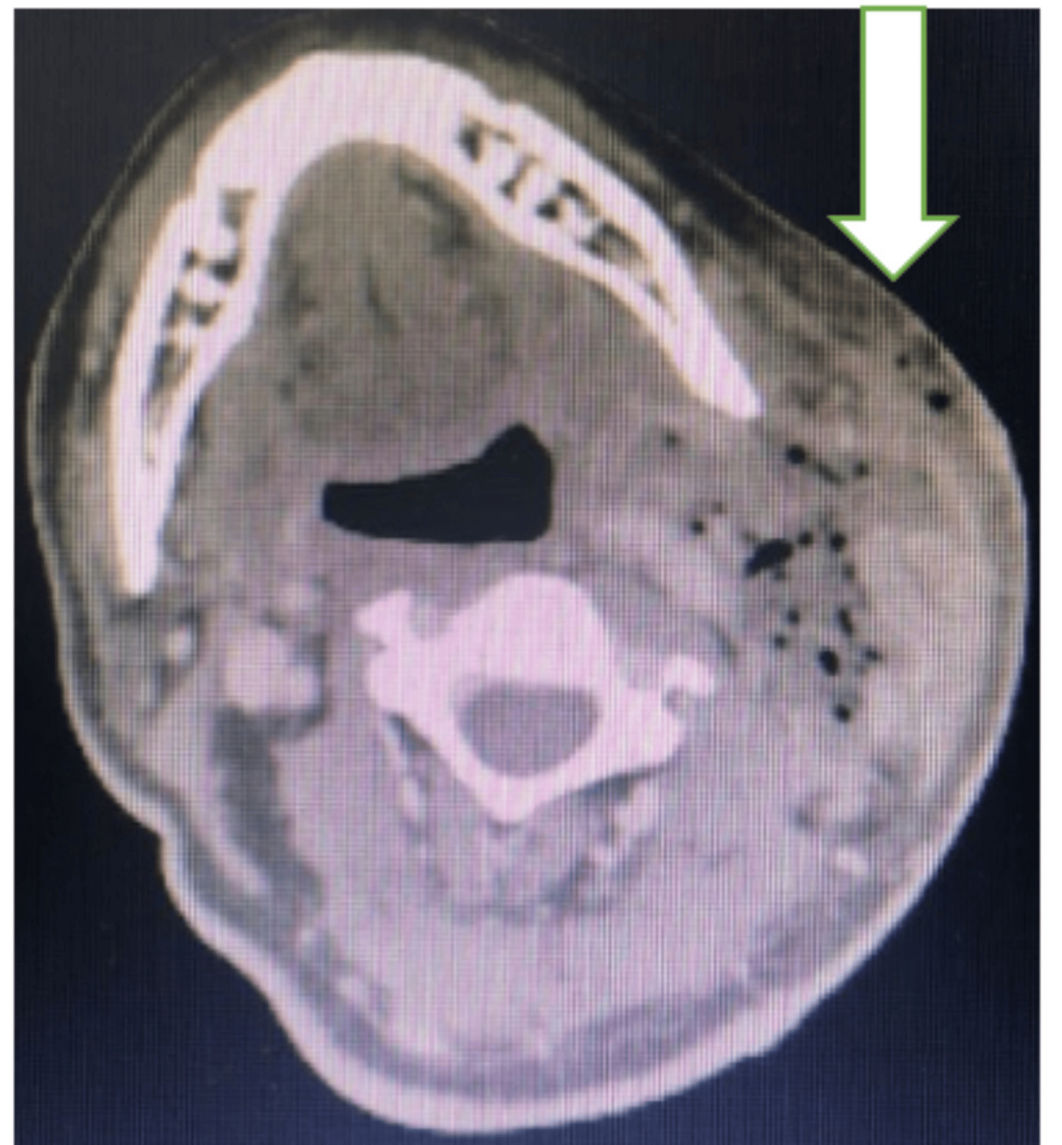
Contrast-enhanced CT showing extensive necrotizing inflammatory changes involving the left parotid, parapharyngeal, submandibular, and carotid spaces. The white arrow indicates the necrotic collection lateral to the sternocleidomastoid muscle.

Contrast-enhanced computed tomography (CT) of the neck, performed on the day of admission, demonstrated extensive inflammatory changes involving the left parotid, parapharyngeal, submandibular, and carotid spaces (Figure 2). A sizeable necrotic collection measuring 17 × 66 mm was identified lateral to the sternocleidomastoid muscle, extending to the thyroid level.

Initial management

The infectious disease team recommended that the patient be admitted under ear, nose, and throat (ENT) care and started on empirical intravenous cefuroxime and clindamycin for broad-spectrum coverage. Supportive measures included strict blood sugar control, analgesia, and regular oral hygiene using antiseptic mouthwash. Conservative management was chosen initially due to the patient’s stability, but surgery was performed urgently when there was no improvement. Antimicrobial therapy with cefuroxime and clindamycin was started on admission and continued intravenously for three weeks, including after surgery, as Klebsiella pneumoniae was sensitive to the initial regimen.

On day five of admission, ultrasound-guided aspiration was performed to address the necrotic collection, yielding 5 mL of purulent material. Culture results confirmed the presence of Klebsiella pneumoniae, which is sensitive to the initiated antibiotic regimen. Despite the aspiration, the swelling persisted with no significant improvement, prompting further intervention.

Surgical management

**Figure 3:**
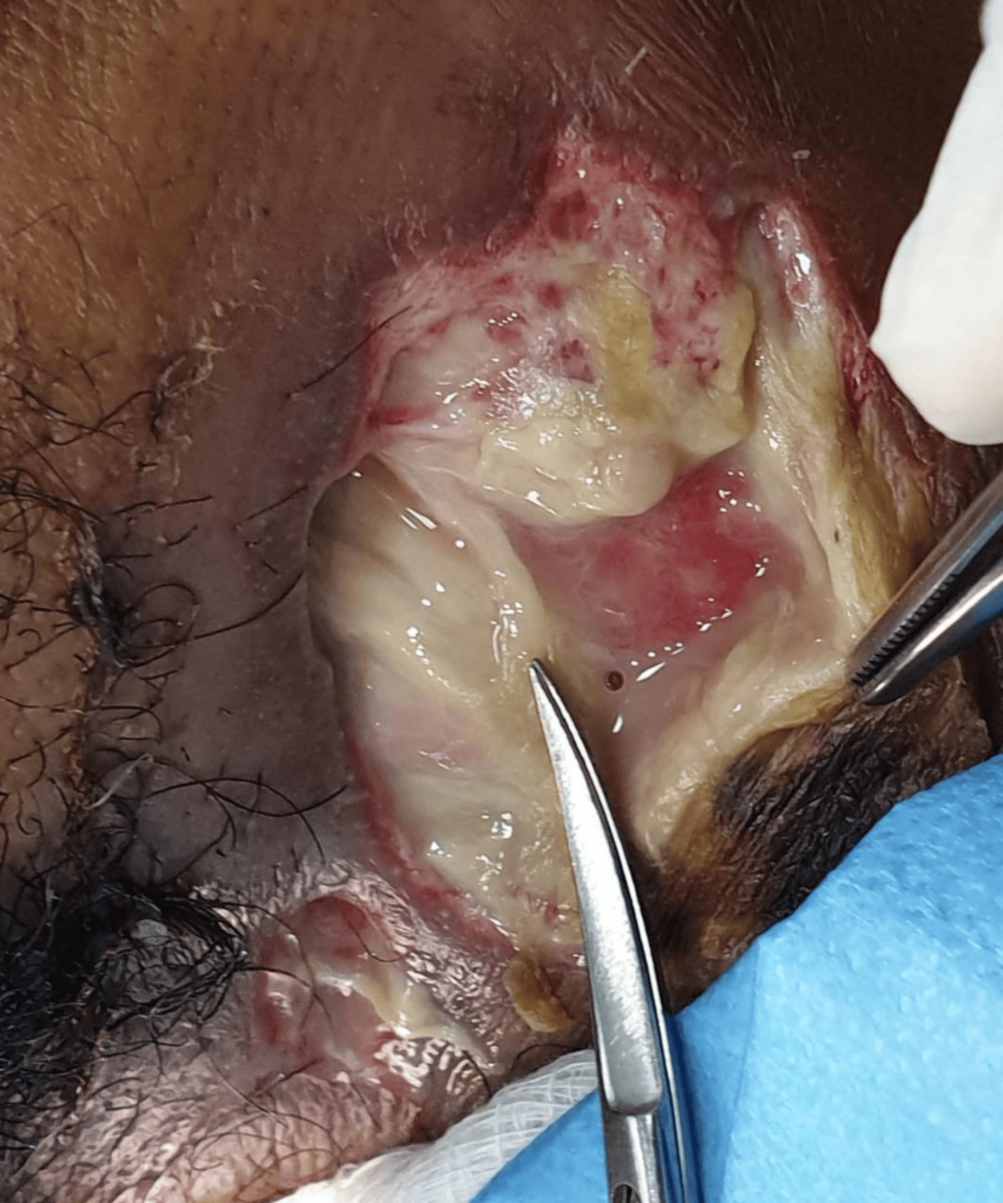
Intraoperative view during debridement showing extensive necrotic tissue in the cervical region.

On day seven, the patient underwent open surgical drainage and debridement under general anesthesia (Figure 3). A transverse cervical incision provided access to the infected region, revealing extensive necrotic tissue involving the submandibular, parapharyngeal, and carotid spaces. The necrotic tissue was excised, and thorough debridement was performed. Hemostasis was achieved, and the wound was left open for regular dressing changes and monitoring.

**Figure 4:**
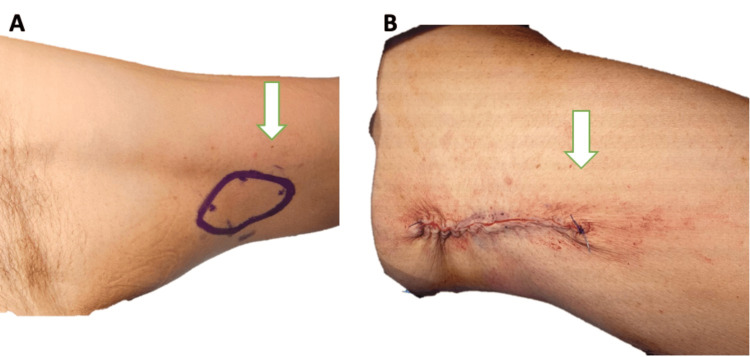
A graft was harvested from the inner left arm, trimmed to fit the defect, and secured with sutures. A: donor site on the inner left arm before graft harvest, indicated by a white arrow; B: closure of the donor site after graft harvest. Images are labeled for clarity, with white arrows highlighting the areas of interest.

**Figure 5:**
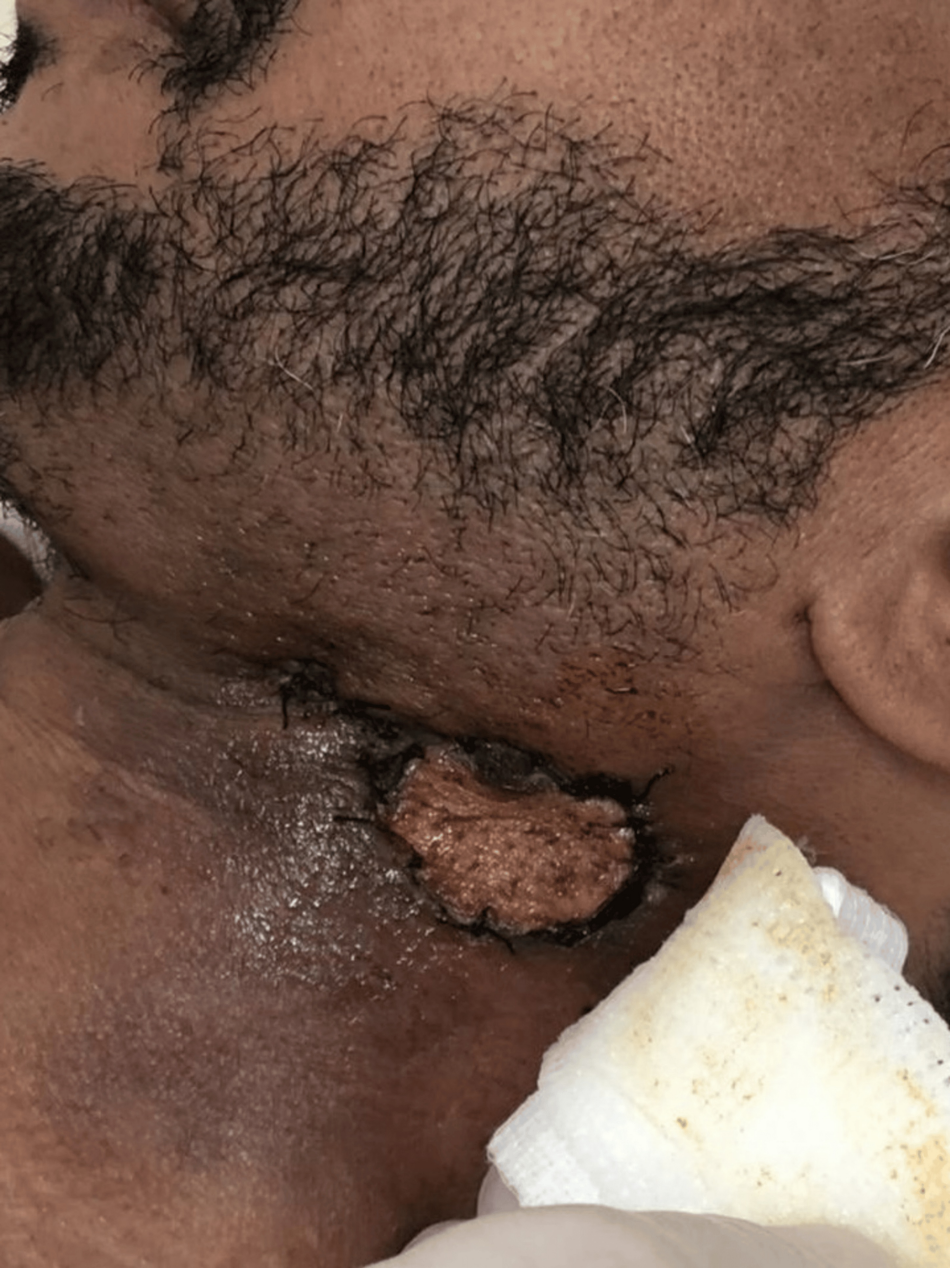
Postoperative image of the wound after suturing the full-thickness skin graft into the defect.

Given the extensive tissue loss, reconstructive surgery was planned. On day 14, the patient underwent full-thickness skin grafting to cover the debrided area. A graft was harvested from the inner left arm, trimmed to fit the defect, and secured with sutures (Figure 4). The donor site was closed primarily, and both sites were dressed appropriately (Figure 5). The patient tolerated the procedure well, with no immediate complications.

Outcome and follow-up

**Figure 6:**
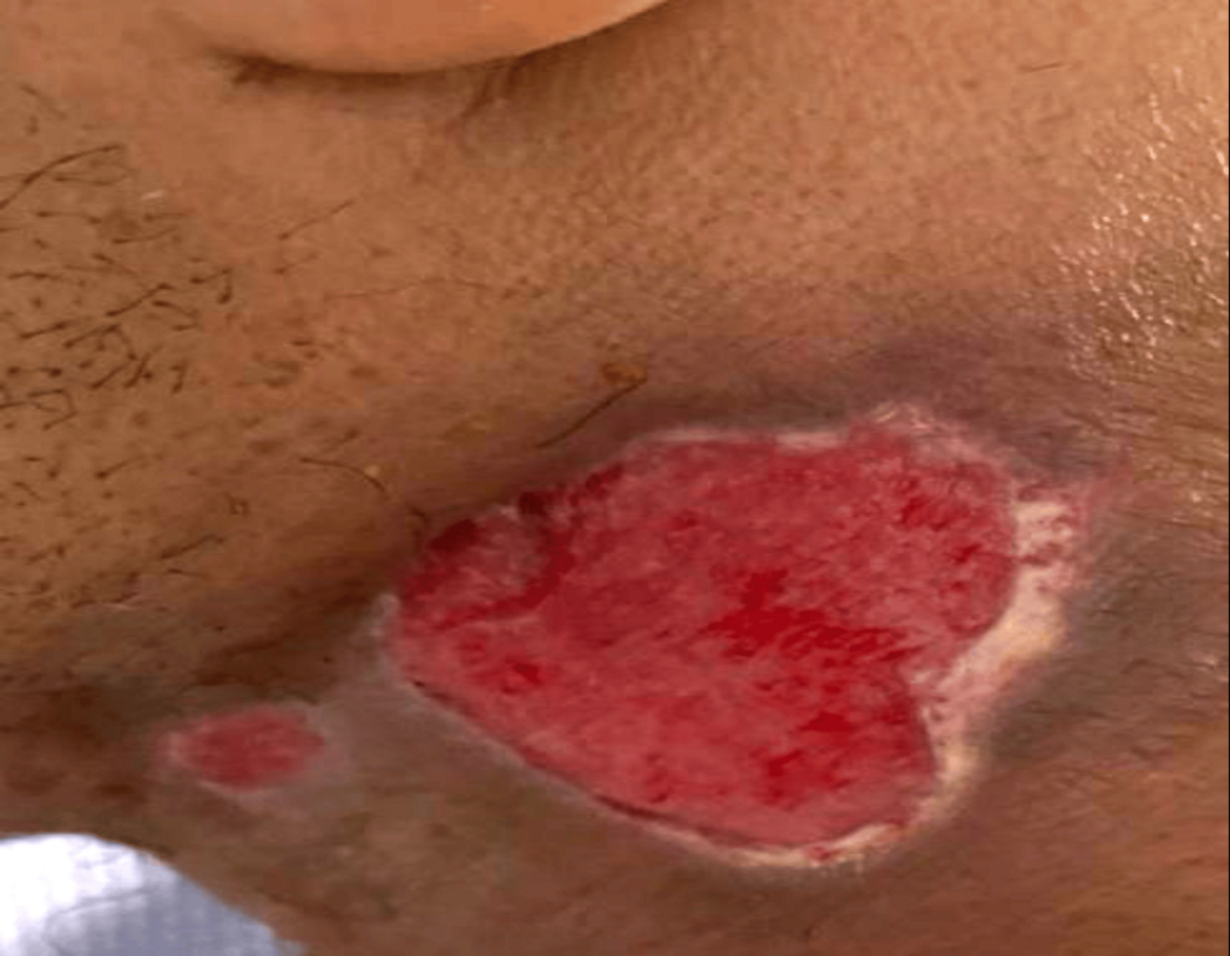
Four-week follow-up showing complete graft integration and restored neck contours with no signs of infection.

The patient showed significant improvement postoperatively, with successful graft integration and resolution of the infection. He was discharged after three weeks with no functional deficits or recurrence (Figure 6). A four-week follow-up confirmed complete recovery, with intact graft viability and restored neck contours.

## Discussion

Necrotizing infections of the neck are exceedingly rare. Due to their rapid progression, the potential for life-threatening complications, and the anatomical complexity of the cervical region, they present unique clinical challenges [4].

Diabetes mellitus, a recognized risk factor, impairs immune responses through hyperglycemia and microvascular damage, facilitating severe infections. This case highlights how even a superficial neck infection in patients with diabetes can rapidly evolve into a life-threatening condition involving multiple cervical spaces [11].

The involvement of Klebsiella pneumoniae, though rare in necrotizing neck infections, significantly complicates this case. Its virulence factors, including hypermucoviscosity and biofilm formation, facilitate rapid tissue invasion and immune evasion, making treatment more challenging [11,12]. Gunnarsson GL et al. (2009) reported a case of necrotizing fasciitis caused by Klebsiella pneumoniae in a 77-year-old patient with diabetes with cervical swelling. Despite surgical debridement and antibiotics, the patient suffered significant morbidity due to tissue destruction [13]. Rahim GR et al. have highlighted that Klebsiella pneumoniae-induced necrotizing fasciitis is becoming more common, particularly among patients with diabetes. This is attributed to the bacterium's virulence factors and the immunosuppressed state often seen in diabetes. Immediate surgical intervention is critical for improving outcomes, as emphasized by their review [14]. In contrast, the current case highlights the importance of a multidisciplinary approach and advanced reconstructive techniques for achieving optimal recovery.

Puncture drainage in necrotizing fasciitis is useful for culture but unlikely to improve the condition. Immediate surgery is critical when signs like redness, gas, and systemic involvement are present. Prompt diagnosis and decisive action are essential for better outcomes in such cases.

Infections in the neck can rapidly spread through fascial planes to nearby spaces, such as the mediastinum, potentially causing life-threatening complications. Diagnosing these infections is challenging due to early symptoms that are often vague and resemble less severe conditions, such as cellulitis or superficial abscesses [7].

In this case, contrast-enhanced CT played a critical role in delineating the extent of the infection, identifying necrotic collections, and guiding both initial aspiration and subsequent surgical debridement. The successful management of this case highlights the indispensable role of advanced imaging in diagnosing necrotizing infections and planning their treatment [1].

Aspiration of necrotic collections aids diagnosis and therapy. While insufficient alone, ultrasound-guided aspiration provides microbiological confirmation, reduces the bacterial load, and supports multidisciplinary management.

Aggressive surgical intervention remains the cornerstone of treatment for necrotizing infections. Early and thorough debridement of necrotic tissue is essential to controlling the disease, halting its progression, and preventing systemic complications [15]. Reconstruction following extensive debridement is equally critical, particularly in a functionally and cosmetically sensitive area like the neck. In this case, a full-thickness skin graft was successfully used to restore tissue integrity and minimize donor-site morbidity, resulting in a favorable aesthetic and functional outcome.

A key limitation of this case was the delayed surgical intervention, as early debridement is the gold standard for necrotizing infections. Earlier surgery could have minimized complications and expedited recovery.

This case underscores several key lessons. Early recognition and intervention are paramount in managing necrotizing infections, particularly in high-risk populations like patients with diabetes. Maintaining a high index of suspicion for medical conditions that can cause neck swelling and pain is essential. Contrast-enhanced CT is invaluable for assessing infection extent and guiding treatment.

Tailored management strategies are vital for optimizing outcomes, including targeted antibiotic therapy and advanced surgical techniques like extensive debridement and full-thickness skin grafting. Together, these insights provide a framework for clinicians to manage similarly complex and rare infections effectively [8].

Finally, the patient’s successful recovery emphasizes the value of comprehensive and coordinated care by ENT, infectious disease, radiology, and surgical teams to achieve optimal outcomes, even in the most challenging scenarios.

## Conclusions

This case underscores the critical need for vigilance in diagnosing necrotizing neck infections, particularly in patients with diabetes. Early diagnosis, targeted antibiotics, and timely surgical intervention are essential for successful outcomes. The collaborative efforts of ENT, infectious disease, radiology, and surgical teams exemplify the importance of a multidisciplinary approach in managing such complex conditions. By documenting this rare Klebsiella-induced necrotizing infection, this report provides valuable guidance for clinicians and highlights the efficacy of aggressive, team-based management in overcoming life-threatening complications.
